# Human adipose-derived mesenchymal stem cells attenuate collagen antibody-induced autoimmune arthritis by inducing expression of FCGIIB receptors

**DOI:** 10.1186/s12891-015-0634-y

**Published:** 2015-07-27

**Authors:** Hyoju Yi, Kwi Young Kang, Youngkyun Kim, Hyerin Jung, Yeri Alice Rim, Narae Park, Juryun Kim, Seung Min Jung, Sung-Hwan Park, Ji Hyeon Ju

**Affiliations:** CiSTEM Laboratory, Convergent Research Consortium for Immunologic Disease, Seoul St. Mary’s Hospital, College of Medicine, The Catholic University of Korea, Seoul, Korea; Division of Rheumatology, Department of Internal Medicine, Seoul St. Mary’s Hospital, College of Medicine, The Catholic University of Korea, 505 Banpo-dong, Seocho-gu, 137-040 South Korea

**Keywords:** Adipose-derived stem cell, Mesenchymal stem cell, Collagen antibody-induced arthritis, FC gamma receptors

## Abstract

**Background:**

Adipose-derived stem cells (ASCs) are mesenchymal stem cells (MSCs) derived from adipose tissue. MSCs have multiple properties including anti-inflammatory and immunomodulatory effects in various disease models and human diseases. However, the mechanisms underlying this wide range of effects need to be explored.

**Methods:**

Collagen antibody-induced arthritis (CAIA) is a unique model in which arthritis is rapidly and strongly induced. ASCs were intraperitoneally infused into CAIA mice before or after arthritis induction. The serum levels of various cytokines, adipokines, and chemokines were measured. The expression of FC gamma receptors (FCGRs) was investigated in peritoneal macrophages *ex vivo*. RAW264.7 cells and ASCs were co-cultured to elucidate the direct and indirect role of ASCs on FCGR expression.

**Results:**

ASCs attenuated arthritis in CAIA mice. Serum levels of tumor necrosis factor α, interleukin (IL)-15, resistin, and leptin were reduced in ASC-treated CAIA mice, whereas serum levels of IL-6 and adiponectin were not affected. In peritoneal macrophages isolated from ASC-treated mice, expression of FCGRIIB, which is immunoinhibitory, was higher than that of FCGRI. Co-culture of ASCs with RAW264.7 cells modulated the expression of FCGRs. The expression patterns and timings of peak expression differed among FCGRs. Expression of FCGRIIB was higher and peaked earlier than that of FCGRI. FCGRIII expression was not affected by this co-culture.

**Conclusions:**

This is a study to show that ASCs have anti-arthritic effects in CAIA mice. Modulation of FCGRs by ASCs might be a therapeutic mechanism in this antibody-associated arthritis model.

## Background

Mesenchymal stem cells (MSCs) are cells of a stromal origin that can self-renew and differentiate into various lineages of mesenchymal tissues. Furthermore, MSCs exert profound immunosuppressive effects that are superior to those of all other immunosuppressive cell types [1]. The effects of MSCs on immune cells have mostly been studied using bone marrow-derived MSCs (BM-MSCs). BM-MSCs have widespread effects on innate and adaptive immune cells [2]. BM-MSCs can inhibit CD4+ T-cell proliferation and B-cell differentiation and induce the differentiation of regulatory T-cells (T-reg) [1, 3–5]. In relation to innate immune cells, BM-MSCs suppress the generation of dendritic cells from monocytes [6], reduce the expression of CD80 and CD86 co-stimulatory molecules on antigen-presenting cells (APCs), and reduce the production of pro-inflammatory cytokines, such as interleukin (IL)-2, interferon-γ, and tumor necrosis factor α (TNFα), by APCs [7].

Adipose-derived MSCs (ASCs) and BM-MSCs can both differentiate toward multiple mesodermal tissue types, including bone, cartilage, and adipose tissue, are both immunosuppressive, and have similar surface protein marker expression [8–11]. However, ASCs senesce later than BM-MSCs, which may be beneficial for the treatment of chronic or persistent conditions. ASCs have a multi-lineage differentiation capacity and elicit immunosuppressive effects on activated immune cells [12, 13]. ASCs release growth factors that are important for wound healing, modulate the immune system, decrease inflammation, and home to injured tissues [14]. ASCs may be of great clinical utility in regenerative therapies for Parkinson’s disease, Alzheimer’s disease, spinal cord injury, heart diseases, and rheumatoid arthritis (RA).

The immunosuppressive effects of ASCs are well known. In vitro, ASCs inhibit the proliferation of activated lymphocytes via cell-cell binding and paracrine signaling [15]. Expanded ASCs have immunosuppressive properties in mice, thereby alleviating graft-versus-host-disease and colitis [16, 17]. ASCs also have anti-inflammatory effects via inducing immune tolerance in a RA mouse model, namely, type II collagen-induced arthritis (CIA) mice [16]. The immunosuppressive effects of ASCs in CIA were explained by Th1/Th17 suppression and T-reg induction [16, 18].

RA involves a multicellular inflammatory process, including the infiltration of lymphocytes and granulocytes into articular cartilage, proliferation of synovial fibroblasts and macrophages, and neovascularization of the synovial lining of joints. Many cellular components (macrophages, dendritic cells, neutrophils, T-cells, and B-cells), cell surface molecules, signaling components, and humoral components interact and aid the progression of RA [19]. CIA is the most commonly used arthritis model and is induced by type II collagen treatment. Collagen antibody-induced arthritis (CAIA; induced by anti-type II collagen antibodies (anti-COL II)) is another widely used mouse model [20]. The actions of anti-collagen antibodies are initiated by direct binding to their antigens and involve immune complex formation, immune complex deposition, and activation of complement and Fc receptors [19]. Type II collagen-specific antibodies induce arthritis in the absence of T- and B-cells [21]; therefore, CAIA is considered to be a T-cell- and B-cell-independent arthritis model, in contrast to CIA. The suppressive effects of ASCs on arthritis are related to the T-cell balance in CIA mice, and the effects of ASCs on other immune cells, aside from T-cells, have not been investigated.

This study investigated the effects of human ASCs on autoimmune arthritis in CAIA mice, which is not related to the T-cell immune response, and analyzed the influence of ASC treatment on serum levels of adipokines in vivo.

## Methods

### Animals and ethics

All procedures involving animals were in accordance with the Laboratory Animals Welfare Act, the Guide for the Care and Use of Laboratory Animals, and the Guidelines and Policies for Rodent Experimentation provided by the Institutional Animal Care and Use Committee of the School of Medicine of The Catholic University of Korea. The study protocol was approved by the Institutional Review Board of The Catholic University of Korea (CUMC-2012-0033-01). Female DBA1/J mice (7-week-old; OrientBio, Korea) were purchased and housed in specific pathogen-free conditions under approved institutional guidance.

### Induction of CAIA and injection of ASCs

Seven-week-old female DBA1/J mice (five mice per group) were intravenously injected with anti-CII (2 mg; Chondrex, WA, USA). After 3 days, 50 μg of lipopolysaccharide (LPS; Chondrex) was administered intraperitoneally to mice. As a control, the same volumes of normal saline were injected into wild-type mice instead of anti-CII and LPS (WT group). For the ASC-pre-injected group (ASC pre), 1 × 10^7^ cells/mouse (in 500 μl of saline) were injected intravenously three times every other day starting 3 days prior to immunization with anti-CII. For the ASC-post-injected group (ASC post), ASCs were injected identically starting 4 days after immunization with anti-CII.

### Evaluation of disease severity

The incidence and severity of arthritis were monitored and scored as described previously [22]. The incidence was calculated as the percentage of mice in which one or more paws were swollen.

### Histological evaluation of arthritis

Mice were sacrificed 14 days after immunization with anti-CII. A hind limb of each mouse was removed and fixed in 10 % formalin. After decalcification in 10 % (*w/v*) EDTA, the samples were embedded in paraffin. The 4 μm-thick sections were stained with hematoxylin and eosin (H&E) or toluidine blue. The inflammation score and joint destruction score were determined. Briefly, the presence of synovial hyperplasia and leukocyte infiltration in H&E-stained samples were graded in a blinded fashion by three independent observers; the sum of the two values was used as the inflammation score. The presence of pannus formation and cartilage erosion were scored and the sum of the two scores was used as the joint destruction score.

### Assessment of serum levels of cytokines and hormones

Venous blood (300 μl) was obtained from mice on the day of sacrifice. Blood samples were incubated at room temperature and then centrifuged for 10 min at 4 °C to obtain serum. Serum samples were used to assess cytokines and hormones. Resistin, gastric inhibitory polypeptide (GIP), glucagon-like peptide 1 (GLP-1), ghrelin, leptin, IL-15, IL-1α, IL-6, IL-7, TNFα, and adiponectin were analyzed using Procarta kits from Affymetrix (Fremont, CA, USA) according to the manufacturer’s protocols. All specimens were assayed in triplicate. Multiplex immunoassays were performed using the Luminex 100 IS System (Luminex Corp., Austin, TX, USA). Sample concentrations were calculated from standard curves using Bio-Plex Manager 4.1.1 (Bio-Rad Laboratories, Hercules, CA, USA).

### Cells

The murine macrophage cell line RAW264.7 was cultured in Dulbecco’s modified Eagle’s medium (DMEM; Gibco) supplemented with 10 % fetal bovine serum (FBS; Gibco) and 1 % penicillin/streptomycin solution (P/S solution; Gibco). ASCs (passage 2–3) were purchased from Catholic MASTER Cells (Seoul, Korea) and maintained in DMEM supplemented with 10 % FBS and 1 % P/S solution.

### Isolation of peritoneal cells

Five milliliters of phosphate-buffered saline containing 3 % FBS was injected per mouse into the peritoneal cavity using a 10-ml syringe. After gentle massage, the peritoneal fluid was collected using the same syringe and centrifuged at 1500 × g for 5 min. The isolated cells were used for RNA purification.

### Reverse-transcription PCR

Total RNA was purified from cells using TRIzol reagent (Invitrogen) following the manufacturer’s instruction. A Revertaid^™^ First Stranded cDNA Synthesis Kit (Fermentas) was used to synthesize cDNA from RNA. PCR was performed using an iTaq DNA Polymerase Kit (iNtRON biotechnology) following the manufacturer’s guidelines. The primer sequences used for PCR are as follows: 5’- TGCGGAACCAGAGCAGGGGT-3’ and 5’- TTTGGGCCAGTGTTCCCGCC-3’ for FCGRI (NM_010186.5), 5’- TGGGAGGTCCATCCGGAGCC-3’ and 5’- TCAGGAGGATTGTCTGGAACCTGC-3’ for FCGRIIB (NM_010187.2), 5’-TTCCACCACTGACAATTCTGCTGCT-3’ and 5’-GGCCCGTGTCCACTGCAAACA-3’ for FCGRIII (NM_010188.5), and 5’-GCCAAACGGGTCATCATCTC-3’ and 5’-GACACATTGGGGGTAGGAAC-3’ for GAPDH (NM_008084.2).

### Statistical analysis

Statistical analysis was performed using Student’s *t*-test. *p* < 0.05 was considered significant.

## Results

### ASCs ameliorate arthritis in CAIA mice

**Fig. 1:**
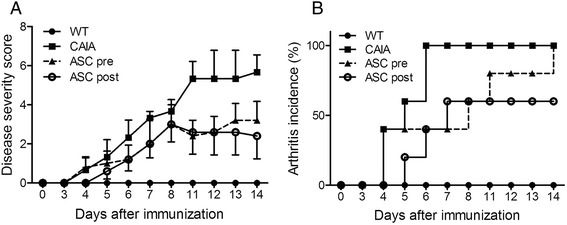
Injection of adipose-derived mesenchymal stem cells (ASCs) ameliorates arthritis in collagen antibody-induced arthritis (CAIA) mice. CAIA mice were injected with ASCs three times every other day starting 3 days prior to (ASC pre group) or 4 days after (ASC post group) immunization with an anti-collagen type II antibody. As controls, wild-type mice were injected with saline (WT group) and CAIA mice were similarly immunized but not injected with ASCs (CAIA group). Each group comprised 5 mice. The disease severity score (**a**, mean ± standard error of the mean) and incidence of arthritis (**b**) were calculated at various numbers of days following immunization

To investigate the effects of ASCs on CAIA, 1 × 10^7^ ASCs were injected three times into CAIA mice. The timing of ASC injection might be important; therefore, we divided ASC-injected mice into two groups: ASC pre and ASC post. In the ASC pre group, ASCs were injected before arthritis symptoms arose. In the ASC post group, ASCs were injected after LPS boosting. Both ASC-injected groups had an ameliorated disease severity score and exhibited delayed disease incidence (Fig. 1a and b).

### ASCs prevent inflammation and bone destruction

**Fig. 2:**
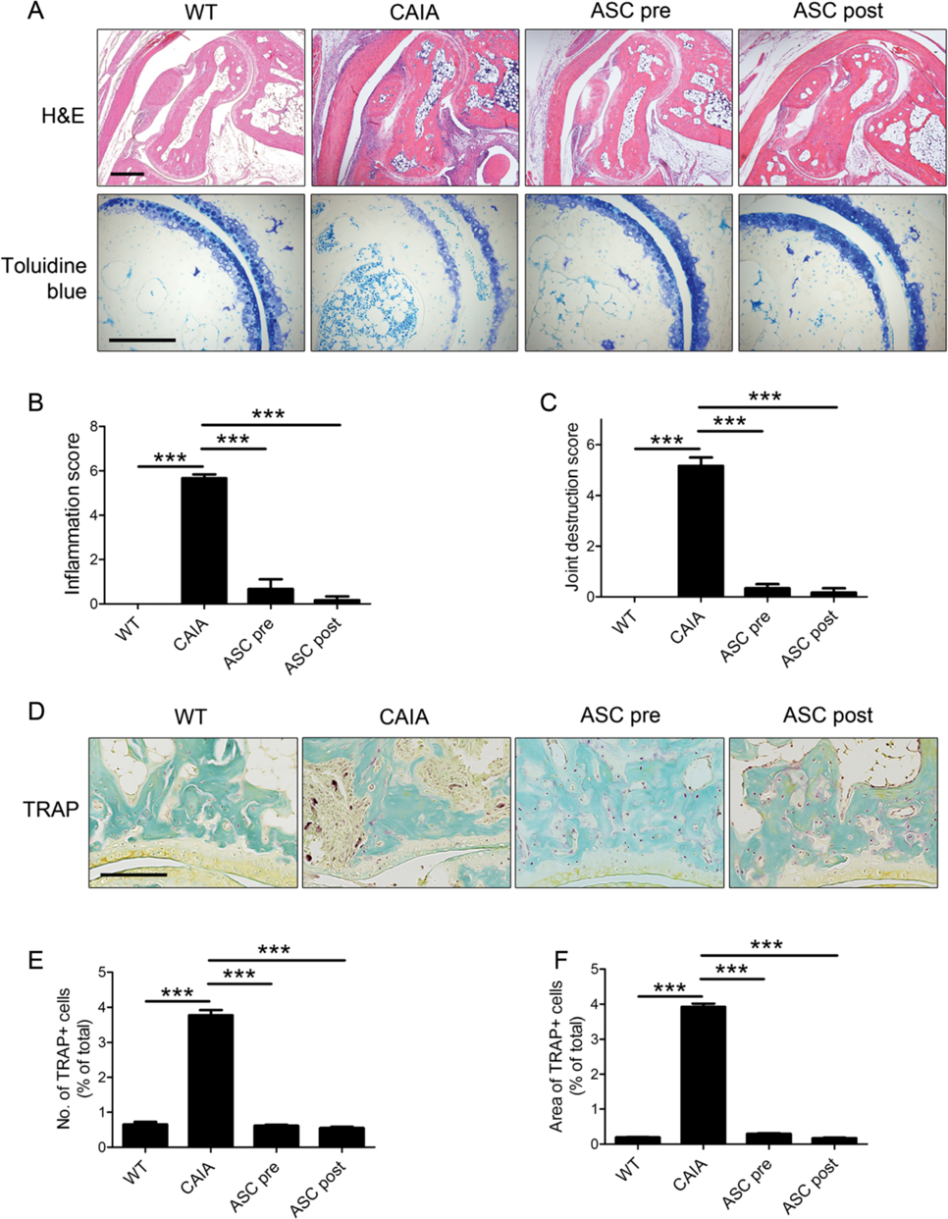
Injection of adipose-derived mesenchymal stem cells (ASCs) prevents inflammation and bone destruction. Collagen antibody-induced arthritis (CAIA) mice were injected with ASCs three times every other day starting 3 days prior to (ASC pre group) or 4 days after (ASC post group) immunization with an anti-collagen type II antibody. As controls, wild-type mice were injected with saline (WT group) and CAIA mice were similarly immunized but not injected with ASCs (CAIA group). Each group comprised five mice. **a** Hind limbs were histologically evaluated by staining with hematoxylin and eosin (H&E; scale bar, 400 μm) or toluidine blue (scale bar, 200 μm) at 14 days after immunization. From this, the inflammation score (**b**) and joint destruction score (**c**) were determined. **d** Hind limbs were histologically evaluated by staining with TRAP at 14 days after immunization. From this, the percentage of TRAP-positive cells (**e**) and the area of these cells (**f**) were determined. All quantitative data show the mean ± standard error of the mean. *** *p* < 0.001 using Student’s *t*-test

The effects of ASCs were examined histologically by H&E and toluidine blue staining (Fig. 2a). The histological inflammation and bony destruction scores were lower in the ASC-injected groups than in control CAIA mice (Fig. 2b and c). TRAP staining showed that multinucleated TRAP-positive osteoclasts (>3 nuclei) were more prominent in the joints of control CAIA mice than in the joints of mice in the ASC pre and ASC post groups (Fig. 2d). The number and area of osteoclasts were higher in the joints of control CAIA mice than in the joints of mice in the ASC pre and ASC post groups (Fig. 2e and f).

### Serum levels of TNFα, IL-15, resistin, and leptin are decreased in ASC-injected mice

**Fig. 3:**
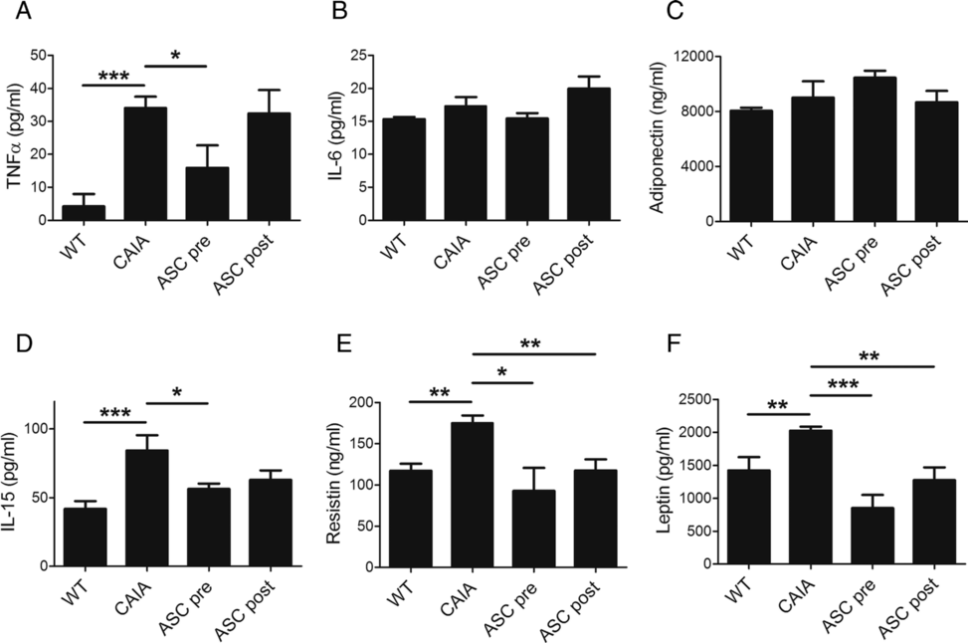
Serum levels of various cytokines/hormones are reduced in adipose-derived mesenchymal stem cell (ASC)-injected mice. Collagen antibody-induced arthritis (CAIA) mice were injected with ASCs three times every other day starting 3 days prior to (ASC pre group) or 4 days after (ASC post group) immunization with an anti-collagen type II antibody. As controls, wild-type mice were injected with saline (WT group) and CAIA mice were similarly immunized but not injected with ASCs (CAIA group). Each group comprised five mice. Serum levels of tumor necrosis factor α (TNFα, **a**), interleukin (IL)-6 **b**, adiponectin **c**, IL-15 **d**, resistin **e**, and leptin (**f**) were evaluated using multiplex immunoassays at 14 days after immunization, the day on which mice were sacrificed. Data show the mean ± standard error of the mean. * *p* < 0.05, ** *p* < 0.01, and *** *p* < 0.001 using Student’s *t*-test

To understand the mechanism underlying the effects of ASCs on CAIA, we examined the serum levels of five cytokines (IL-15, IL-1α, IL-6, IL-7, and TNFα), five diabetes-related hormones (resistin, GIP, GLP-1, ghrelin, and leptin), and adiponectin using multiplex immunoassays. The serum levels of IL-15, resistin, and leptin were decreased in the ASC pre and ASC post groups (Fig. 3d, e, and f). The serum level of TNFα was reduced in the ASC pre group, but not in the ASC post group (Fig. 3a). IL-6 and adiponectin did not significantly differ among the experimental groups (Fig. 3b and c).

### ASCs induce the over-expression of Fc gamma receptors (FCGRs) in peritoneal cells

**Fig. 4:**
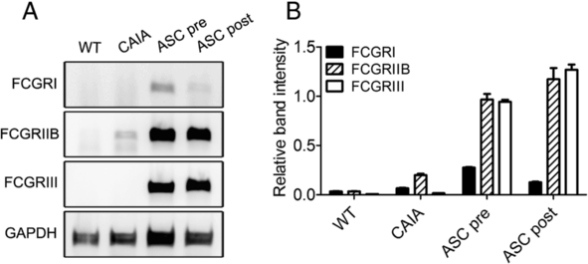
Adipose-derived mesenchymal stem cells (ASCs) increase expression of Fc gamma receptors (FCGRs) in peritoneal cells. Collagen antibody-induced arthritis (CAIA) mice were injected with ASCs three times every other day starting 3 days prior to (ASC pre group) or 4 days after (ASC post group) immunization with an anti-collagen type II antibody. As controls, wild-type mice were injected with saline (WT group) and CAIA mice were similarly immunized but not injected with ASCs (CAIA group). Each group comprised 5 mice. Peritoneal cells were isolated at sacrifice time. Total RNA was extracted from these cells and reverse-transcription PCR was performed with primers designed to amplify FCGRI, FCGRIIB, FCGRIII, and GAPDH (**a**). The intensities of the bands, relative to those of GAPDH, were determined (**b**, mean ± standard error of the mean)

T-cells and B-cells are rarely involved in disease pathogenesis in CAIA models; therefore, the immune complex is regarded as a potential player in this system. The immune complex usually signals via FCGRs. The relationship between FCGRs and stem cells has not been explored. The mRNA levels of FCGRs found on macrophages were investigated. Peritoneal cells of ASC-injected mice expressed FCGRI, FCGRIIB, and FCGRIII (Fig. 4a). Expression of FCGRIIB, which is a well-known anti-inflammatory signal mediator, was higher than that of FCGRI (Fig. 4b).

### The conditioned media of ASCs induces the over-expression of FCGRI and FCGRIIB in macrophages

**Fig. 5:**
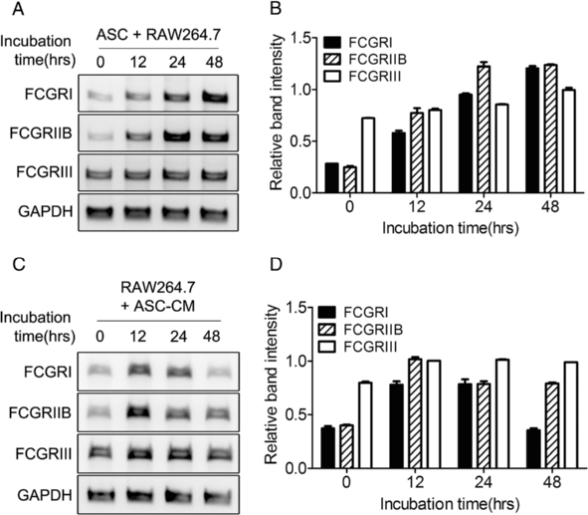
Adipose-derived mesenchymal stem cell (ASCs) conditioned media increases Fc gamma receptor (FCGR) expression in macrophages. RAW264.7 murine macrophages were co-cultured with commercially purchased ASCs (**a** and **b**) or with the conditioned media of these cells (ASC-CM, **c** and **d**) for various numbers of hours. Total RNA was extracted from the cells and reverse-transcription PCR was performed with primers designed to amplify FCGRI, FCGRIIB, FCGRIII, and GAPDH (**a** and **c**). The intensities of the bands, relative to those of GAPDH, were determined (**b** and **d**, mean ± standard error of the mean)

To simulate the effects of ASCs on FCGRs, ASCs and RAW264.7 cells were co-cultured. Co-culture with ASCs increased the expression of FCGRI and FCGRIIB in a time-dependent manner (Fig. 5a). Although FCGRI expression increased concomitant with that of FCGRIIB, expression of FCGRIIB increased more than expression of FCGRI, which has a pro-inflammatory role (Fig. 5b).

The conditioned media of ASCs increased expression of FCGRI and FCGRIIB in RAW264.7 cells (Fig. 5c). FCGRI and FCGRIIB levels were highest at 12 hr after stimulation with this conditioned media. Thereafter, expression of FCGRs decreased in a time-dependent manner. In conclusion, expression of FCGRI and FCGRIIB on macrophages was associated with ASCs or ASC-secreted molecules. The higher expression of FCGRIIB than of FCGRI is an interesting phenomenon that may be linked with the anti-inflammatory properties of ASCs (Fig. 5d).

## Discussion

The present study demonstrates that human ASCs can induce expression of inhibitory FCGRs on macrophages in CAIA mice. This is a novel mechanism via which ASCs elicit immunosuppressive effects.

There are several mechanisms through which ASCs suppress immunity. ASCs secrete transforming growth factor β1, a growth factor that promotes premature immune tolerance [8]. Furthermore, ASC treatment increases expression of IL-10, which might enhance T-reg function, in mice with experimental colitis. In vitro, human ASCs suppress the collagen-specific response of T-cells from patients with RA. Human ASCs inhibit the proliferative response of collagen-activated T-cells, as well as the production of inflammatory cytokines by these cells, and increase the number of IL-10-producing T-cells and monocytes. Human ASCs also stimulate the generation of T-reg [23]. Human ASCs have immunosuppressive effects in CIA mice [16, 18]. Systemic infusion of human ASCs reduces the incidence and severity of CIA, decreases collagen-specific Th1/Th17 cell differentiation, and induces T-reg generation.

CIA is an immunologically mediated disease involving T-cells, B-cells, and inflammatory cells that infiltrate joints, whereas CAIA is a T-cell- and B-cell-independent arthritis model [21]. In the present study, ASC treatment in CAIA mice significantly decreased the severity of arthritis, but had little effect on the Th17/T-reg balance, which is a mechanism via which ASCs elicit anti-arthritic effects in CIA. This may be related to the T-cell-independent characteristic of CAIA. Our data elicited a novel mechanism in which ASCs elicit immunomodulatory effects by regulating FCGRs.

FCGRs play a crucial role in antibody-mediated autoimmune diseases. FCGRs can mediate various functions, such as antibody-dependent cellular cytotoxicity and phagocytosis. FCGRs are considered to be important immune regulators linked to autoimmunity. The actions of FCGRs are mediated by activating and inhibitory receptors that bind to the same Fc portion of IgG. FCGRI and FCGRIII are activating receptors. FCGRIIb1 and FCGRIIb2 contain an immunoreceptor tyrosine-based inhibition motif that is responsible for mediating inhibitory, rather than activating, functions [24].

FCGRIIB is widely expressed on cells of the innate immune system, including mast cells, eosinophils, basophils, monocytes, neutrophils, dendritic cells, and macrophages. FCGRIIB can modulate the activities of innate immune effector cells [25]. Inhibitory FCGRIIB contributes to immune protection in two ways: (1) by down-regulating effector cell responses, and (2) by maintaining peripheral tolerance [26].

In RA, the balance between activating and inhibitory FCGR signaling controls inflammation and tissue damage [27, 28]. FCGRIIB-deficient mice display a strongly augmented IgG anti-collagen II humoral response that causes a more severe arthritis phenotype than that observed in control mice [29]. Antibodies mediate pro- and anti-inflammatory activities via engagement of their Fc fragment with FCGRs. Mice lacking the common FCGR chain are highly resistant to CAIA. The absence of FCGRIIB in mice exacerbates arthritis [30]. Expression of FCGRs is dysregulated in macrophages of arthritic mice; expression of activating FCGRI and FCGRIII are prolonged and expression of inhibitory FCGRII is down-regulated, resulting in chronic inflammation and severe cartilage destruction [27].

Our data suggest that ASCs elicit inhibitory effects on autoimmune arthritis by controlling FCGR expression on macrophages. Inhibition of macrophages by ASCs reduced the secretion of inflammatory cytokines, such as IL-15, IL-6, and TNF-α. IL-15 is a pro-inflammatory cytokine that is over-expressed in RA and is produced by differentiated macrophages, dendritic cells, and bone marrow stromal cells [31]. IL-6 and TNF-α are well-known pro-inflammatory cytokines that are important to the pathogenesis of RA. Suppression of these pro-inflammatory cytokines by ASCs decreased the severity of arthritis in mice. This is the first report of the inhibitory effect of ASCs on CAIA via increased expression of FCGRIIB. However, the mechanisms underlying the induction of FCGRs by ASCs were not addressed in this study. It remains to be established whether ASCs act directly on FCGR expression.

The results of the present study suggest that treatment with ASCs did not increase the serum levels of pro-inflammatory adipokines. The expression of most pro-inflammatory adipokines, including leptin, is increased in obesity and this contributes to a “low grade inflammatory state”, which causes a variety of metabolic aberrations that affect joints and bone [32]. Leptin is an adipokine with pleiotropic actions that regulate food intake, energy metabolism, inflammation, and immunity [33]. A recent study reported that leptin is involved in promoting the pathogenesis, development, and/or progression of RA [34]. Resistin is also associated with increased inflammation and joint destruction in RA patients [22]. Although ASCs reduced the severity of arthritis, they could potentially cause chronic inflammation if they increase the generation of pro-inflammatory adipokines in vivo. Our data showed that ASCs attenuate arthritis without increasing the serum levels of adipokines.

## Conclusions

In summary, the present study shows for the first time that human ASCs attenuate autoimmune arthritis in CAIA. They induce the expression of inhibitory FCGRs on macrophages and inhibit the secretion of pro-inflammatory cytokines without affecting the serum levels of pro-inflammatory adipokines. The regulation of FCGRIIB by ASC treatment will be the focus of therapeutic approaches to treat RA.

## Abbreviations

Anti-COL II: Anti-type II collagen antibody
APC: Antigen-presenting cell
ASC: Adipose-derived mesenchymal stem cell
ASC post: Adipose-derived mesenchymal stem cell-post-injected group
ASC pre: Adipose-derived mesenchymal stem cell-pre-injected group
BM-MSC: Bone marrow-derived mesenchymal stem cell
CAIA: Collagen antibody-induced arthritis
CIA: Collagen-induced arthritis
DMEM: Dulbecco’s modified Eagle's medium
FBS: Fetal bovine serum
FCGR: Fc gamma receptor
GIP: Gastric inhibitory polypeptide
GLP-1: Glucagon-like peptide 1
H&E: Hematoxylin and eosin
IL: Interleukin
LPS: Lipopolysaccharide
MSC: Mesenchymal stem cell
P/S solution: Penicillin/streptomycin solution
RA: Rheumatoid arthritis
T-reg: Regulatory T-cell
TNFα: Tumor necrosis factor α
